# Risk factors for lumbar intervertebral disc height narrowing: a population-based longitudinal study in the elderly

**DOI:** 10.1186/s12891-015-0798-5

**Published:** 2015-11-09

**Authors:** Koji Akeda, Tomomi Yamada, Nozomu Inoue, Akinobu Nishimura, Akihiro Sudo

**Affiliations:** Department of Orthopaedic Surgery, Mie University Graduate School of Medicine, 2-174 Edobashi, Tsu City, Mie 514-8507 Japan; Department of Clinical Epidemiology and Biostatistics, Graduate School of Medicine, Osaka University, 2-2 Yamadaoka, Suita City, Osaka 565-0871 Japan; Department of Orthopedic Surgery, Rush University Medical Center, 1611 West Harrison Street, Orthopedic Building 205 J, Chicago, 60612 Illinois USA

## Abstract

**Background:**

The progression of disc degeneration is generally believed to be associated with low back pain and/or degenerative lumbar diseases, especially in the elderly. The purpose of this study was to quantitatively evaluate changes in lumbar disc height using radiographic measurements and to investigate risk factors for development of disc height narrowing of the elderly.

**Methods:**

From 1997 to 2007, 197 village inhabitants at least 65 years-old who participated in baseline examinations and more than four follow-up examinations conducted every second year were chosen as subjects for this study. Using lateral lumbar spine radiographs of each subject, L1-L2 to L5-S1 disc heights were measured. The subjects were divided into two groups according to the rate of change in disc height: mildly decreased (≤20 % decrease) and severely decreased (>20 % decrease). A stepwise multiple logistic regression analysis was used to select those factors significantly associated with disc height narrowing.

**Results:**

Disc height at each intervertebral disc (IVD) level decreased gradually over ten years (*p* < 0.01, an average 5.8 % decrease of all disc levels). There was no significant difference in the rate of change in disc height among the IVD levels. Female gender, radiographic knee osteoarthritis and low back pain at baseline were associated with increased risk for disc height narrowing.

**Conclusions:**

We conducted the first population-based cohort study of the elderly that quantitatively evaluated lumbar disc height using radiographic measurements. The risk factors identified in this study would contribute to a further understanding the pathology of disc degeneration.

## Background

With the advent of a populous aging society resulting from increasing life expectancy, the public health issues of the burden of disease and disability have gained prominence [1]. In particular, disability from musculoskeletal disorders in the aging population directly affects quality of life [2–5]. Degenerative disc diseases, or spondylosis of the lumbar spine, is a major factor affecting disability among the elderly [3, 6–8].

Lumbar degenerative disorders, such as spondylosis, lumbar canal stenosis and degenerative spondylolisthesis, are highly prevalent in the elderly [9]. It is generally thought that degenerative lumbar disorders occur during the progression of lumbar disc degeneration [10, 11]. Clinically, disc-space narrowing on lumbar radiographs is a common indicator for intervertebral disc (IVD) degeneration. To date, there are only a few epidemiologic population-based studies examining lumbar disc degeneration, especially in older populations [3, 9, 12, 13]. There are also only a few reports of longitudinal studies on the progression of lumbar disc degeneration [14–17]. In many epidemiologic studies, the extent of lumbar disc degeneration was semi-quantitatively evaluated using lumbar radiographs, but no study has been reported in which lumbar disc height was quantitatively evaluated.

The purposes of this population-based cohort study were to quantitatively evaluate the change and rate of progressive disc degeneration by radiographic measurements of lumbar disc height and to identify risk factors for the development of disc height narrowing in the elderly.

## Methods

### Participants

Data were analyzed from a population-based longitudinal prospective study of osteoporosis and knee osteoarthritis (OA) in a typical mountain village, Miyagawa, in the central Mie Prefecture of Japan [18–20]. A medical examination of village inhabitants at least 65 years-old had been conducted every second year since 1997. This study was conducted with approval of the Committee for the Ethics of Human Research of Mie University and all subjects provided written informed consent before enrollment in the study.

### Clinical interview and physical examination

Subjects completed an interviewer-administrated questionnaire that included information on age, sex, work history, smoking history and medical history, including the presence of knee and/or low back pain. Histories of treatment for rheumatoid arthritis, osteoporosis, kidney and liver disorders, heart disease, hypertension, gout, thyroid disease, tuberculosis, and malignant tumors were recorded. Anthropometric measurements included body height, body weight, body mass index (BMI) and bone mineral density (BMD). The BMD of the forearm was measured using dual energy x-ray absorptiometry (DCS-600EX, Aloka, Tokyo). Anteroposterior radiographs of both knees were graded for radiographic knee OA using the Kellgren-Lawrence (KL) grading system. Definite radiographic knee OA was defined as KL grade 2 or higher. Vertebral compression fractures and calcification of the abdominal aorta were evaluated using lateral thoracic and lumbar radiographs. All radiographs were independently evaluated by three certified orthopedic surgeons who were blinded to the classification groups of this study.

### Radiographic measurements of the lumbar spine

**Fig. 1:**
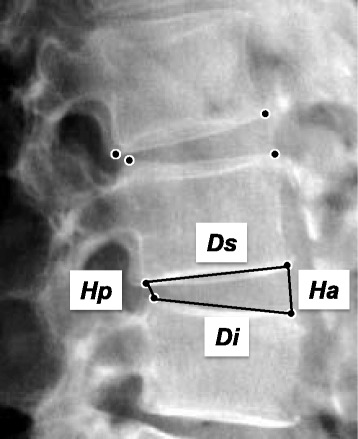
Disc height measurement. *Ha*: anterior disc height, *Hp*: posterior disc height, *Ds*: superior disc depth, *Di*: inferior disc depth. Disc height is expressed as the disc height index (DHI), which was calculated as: [(*Ha* + *Hp*)/(*Ds* + *Di*)] × 100

At each biannual examination, lateral lumbar spine radiographs of each subject were centered on the L3 vertebrae with the subjects in the left lateral recumbent position. The radiographs were digitalized using a scanner (ES-2200, EPSON, Tokyo, Japan). Anterior and posterior disc heights and IVD depth were measured as previously reported [21]. The criterion for positioning landmarks for measuring the discs was that the marks be on the extreme anterior and posterior margins of the vertebral end-plates. A trained observer assessed all lumbar radiographs from L1-L2 to L5-S1 discs using a custom software program in Microsoft Visual C++ with Microsoft Foundation Class programming environment (Microsoft Corp., Redmond, WA). Anterior disc height (Ha), posterior disc height (Hp), superior disc depth (Ds) and inferior disc depth (Di) were measured (Fig. 1). Disc height was expressed as the disc height index (DHI), based on the method of Farfan [22, 23] with modifications, calculated as: [(Ha + Hp)/(Ds + Di)] × 100. Within-observer reproducibility was assessed by test-retest analysis of 40 randomly selected radiographs from this study.

### Classification of ∆DHI

The ∆DHI (%) was calculated as the rate of change in DHI compared to the baseline, [(DHI at follow ‐ up − DHI at baseline)/DHI at baseline] × 100. According to the value of ∆DHI (the rate of change in disc height) at the final examination, the subjects were divided into two groups: mildly decreased (MD) (∆DHI ≥ −20 %) and severely decreased (SD) (∆DHI ˂-20 %). Based on the classification of multilevel disc degeneration [24], the subjects with multilevel (more than two levels) SD were divided into contiguous SD (CSD) and skipped SD (SSD). Furthermore, the subjects with SSD were grouped into subtypes (I-V) dependent on the location of the severely decreased lumbar disc relative to the lumbar discs that were not severely decreased.

### Statistical analysis

A two-way repeated measures analysis of variance was used to compare the IVD levels, assessed by ∆DHI, with times of examinations. Associations between ∆DHI and disc levels were analyzed using the chi-square test. 

For a statistical analysis to identify the risk of progression of disc height narrowing that included severity of disc height narrowing at all lumbar disc levels, subjects were first divided into ordinal three groups according to the number of discs identified as severely decreased: Group A, no lumbar discs identified as severely decreased; Group B, one or two lumbar discs severely decreased; and Group C, three to five lumbar discs severely decreased. These three groups were then rearranged into two categories as follows: ‘group A and group B+ C’; or ‘group A + B and group C’. A stepwise polytomous logistic regression analysis using a cumulative logit model was subsequently performed to identify the risk factors among the three groups. Potential risk factors analyzed, including age, body weight, body height, BMI, presence of knee and/or low back pain, smoking, osteoporosis, radiographic knee OA and calcification of the abdominal aorta, treatment history of diabetes mellitus, kidney disorder, liver disorder, gout, hypertension, thyroid disorder, tuberculosis and osteoporosis, were assessed using the logistic regression model. Odds ratios were estimated between the two categories, ‘group A and group B + C’ and ‘group A + B and group C’ under the proportional odds assumption. A variance inflation factor (VIF) analysis was performed to evaluate the severity of multicollinearity. Data analyses were performed using SAS Version 9.0 (SAS Institute, North Carolina, USA).

## Results

### Characteristics of subjects

The population of this village was 4196 in 1997, including 1463 inhabitants who were 65 years-old or older. At baseline in 1997, 629 of these elderly inhabitants participated in this study. Over the ten years from 1997 to 2007, a possible total of six medical examinations were conducted. 197 inhabitants (62 males, 135 females, mean age: 70.1 years-old) who participated at baseline and had more than four subsequent examinations were chosen as subjects for this study. Of the 197 subjects, 154 (77.4 %) participated in the second examination (in 1999), 170 subjects (85.4 %) in the third (in 2001), 168 subjects (84.4 %) in the fourth (in 2003), 124 subjects (62.3 %) in the fifth (in 2005) and 103 subjects (51.6 %) in the sixth (in 2007). 36 subjects (18.3 %) were followed-up (until final examination) for 6 years, 60 subjects (30.4 %) for 8 years, and 101 subjects (51.3 %) for 10 years. The average follow-up period was 8.67 ± 1.53 years.

The results of anthropometric measurements are summarized in Table 1. Body height and weight were significantly higher in men than in women (*p* < 0.01), although there was no significant difference in BMI. Results of DHI at baseline are summarized in Table 2. At baseline, there were no significant differences in DHI for each disc level between men and women. Results of clinical interviews and interpretation of radiographs (knee OA and calcification of abdominal aorta) at baseline are summarized in Table 3. The percentage of smokers was significantly higher in men (*p* < 0.01). The prevalence of osteoporosis and radiographic knee OA was significantly higher in women (*p* < 0.01, *p* < 0.05, respectively).

**Table 1:** Anthropometric measurements

	Overall	Men	Women
Number of subjects	197	62	135
Age (years)	70.1 ± 4.5	70.4 ± 4.1	69.9 ± 4.7
Body height (cm)	150.9 ± 7.3	158.0 ± 5.2*	147.7 ± 5.6
Body weight (kg)	52.9 ± 7.5	57.7 ± 6.7*	50.9 ± 7.0
BMI (kg/m^2^)	23.2 ± 2.6	22.9 ± 2.3	23.3 ± 2.7

Anthropometric measurements at baseline are presented as mean ± standard deviation (SD)

*BMI* body mass index

*p < 0.01 vs. men

**Table 2:** Disc height index

Disc level	Overall	Men	Women
L1-L2	21.9 ± 3.8	21.6 ± 3.9	22.1 ± 3.8
L2-L3	24.0 ± 4.5	24.4 ± 4.6	23.7 ± 4.5
L3-L4	26.2 ± 5.5	26.5 ± 5.0	26.0 ± 5.7
L4-L5	26.9 ± 6.5	27.0 ± 6.1	26.8 ± 6.8
L5-S1	27.9 ± 8.2	27.4 ± 8.6	28.0 ± 8.0

Disc height index (DHI) at baseline are presented as mean ± standard deviation (SD). There were no significant differences between men and women in the DHI of each disc level

**Table 3:** Baseline characteristics of subjects

Risk factors	Overall	Men	Women
	(*n* = 197)	(*n* = 62)	(*n* = 135)
Low back pain	97 (49)	30 (48.3)	67 (49.6)
Smoking	27 (13.6)	24 (38.7)*	3 (2.2)
Knee pain	95 (48)	14 (22.6)	61 (45.2)
Osteoporosis	57 (28.8)	5 (8.1)	52 (38.5)*
Diabetes mellitus^a^	7 (3.5)	2 (3.2)	5 (3.7)
Kidney disorder^a^	10 (5.1)	1 (1.6)	9 (6.7)
Liver disorder^a^	8 (4.0)	2 (3.2)	6 (4.4)
Gout ^a^	5 (2.5)	2 (3.2)	3 (2.2)
Hypertension^a^	79 (39.9)	17 (27.4)	62 (45.9)
Thyroid disorder^a^	8 (4.0)	3 (4.8)	5 (3.7)
Tuberculosis^a^	9 (4.5)	2 (3.2)	7 (5.2)
Osteoporosis^a^	27 (13.6)	1 (1.6)	26 (19.3)*
Knee OA	44 (22.2)	14 (22.6)	52 (38.5)**
Calcification of AA	83 (41.9)	29 (46.8)	54 (46.0)

Data represent the number of subjects with the risk factor (percentage of total)

*OA* osteoarthritis, *AA* abdominal aorta

*p < 0.01, **p < 0.05 vs. men or women

^a^treatment history of the disease

### Changes in disc height

**Fig. 2:**
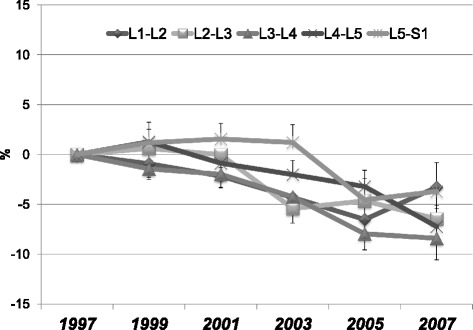
Rate of change in disc height index (∆DHI). The ∆DHI shows a significant gradual decrease over the ten-year period (*p* < 0.0001) (Fig. 3). An analysis of variance revealed no significant disc level and time-point interactions (*p* = 0.63). The ∆DHI at each level shows similar changes over ten years that did not differ significantly among the disc levels (*p* = 0.78)

The DHI at baseline (in 1997) was greatest at L5-S1, decreasing in sequence to L1-L2 (Table 2). The ∆DHI of each disc gradually decreased significantly (*p* < 0.0001) over the ten-year period (Fig. 2). An analysis of variance revealed no significant interaction between disc level and time-point (*p* = 0.63). The ∆DHI at each level had similar changes over ten years and did not differ significantly among the disc levels (*p* = 0.78). The overall average ∆DHI at the final examination (in 2007) was −5.8 ± 2.3 % (L1-L2: −3.2 ± 2.1 %, L2-L3: −6.5 ± 2.1 %, L3-L4: −8.4 ± 2.2 %, L4-L5: −7.3 ± 2.1 %, L5-S1: −3.7 ± 2.3 %). The within-observer reproducibility of DHI, assessed by the intraclass correlation coefficient [ICC], was 0.95.

### ∆DHI classification

**Fig. 3:**
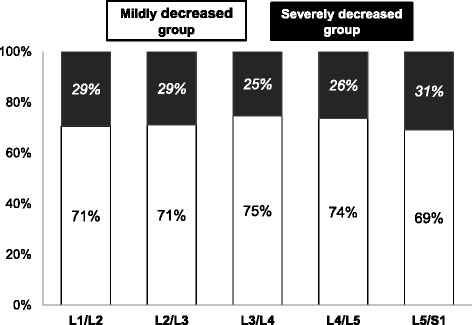
Percentage of discs classified as severely and mildly decreased at each disc level by rate of change in disc height index (∆DHI). At each disc level, the severely decreased group was approximately 30% and the mildly decreased group was approximately 70%

Comparing the ∆DHI classifications at each level, approximately 30 % of the subjects were in the SD group (L1-L2: 29 %, L2-L3: 29 %, L3-L4: 25 %, L4-L5: 26 %, L5-S1: 31 %) and 70 % were in the MD group (L1-L2: 71 %, L2-L3: 71 %, L3-L4: 75 %, L4-L5: 74 %, L5-S1: 69 %) (Fig. 3). There were no significant differences in the percentages of the SD group among the disc levels (*p* = 0.88).

**Fig. 4:**
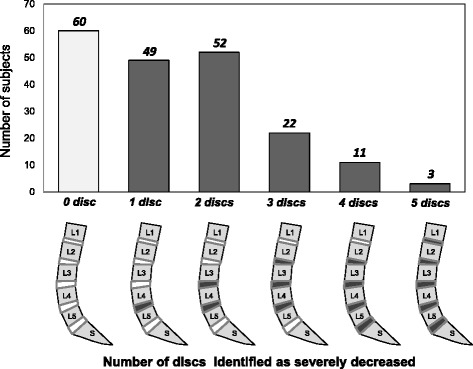
Number of subjects with severely decreased disc height by disc level. The graph shows the number of subjects whose lumbar discs was identified as severely decreased at each level. The illustration below the graph is a representative image of the severely decreased degeneration pattern by number of subjects

The subjects were then sorted according to the number of discs identified as severely decreased (Fig. 4). Within the total number of subjects (*n* = 197), the number of subjects with no lumbar discs severely decreased was 30 % (*n* = 60); with one disc severely decreased was 25 % (*n* = 49); two discs 26 % (*n* = 52); three discs 11 % (*n* = 22); four discs 6 % (*n* = 11); and five discs 2 % (*n* = 3). The number of subjects with one or two discs severely decreased accounted for 55 % of the total.

Among the subjects with multilevel SD (45 % of the total subjects: *n* = 88), contiguous SD (CSD) was 47 % (*n* = 41) and skipped SD (SSD) was 53 % (*n* = 47). Among the subjects with SSD, type I was 60 % (*n* = 28), type II 13 % (*n* = 6), type III 11 % (*n* = 5), type IV 13 % (*n* = 6) and type V 4 % (*n* = 2).

### Prediction of disc height narrowing

Results of the logistic regression analysis are summarized in Table 4. Increased risk for disc height narrowing was significantly associated with female gender (*p* = 0.0006), radiographic knee OA (*p* = 0.0197) and the presence of low back pain (*p* = 0.0253) at baseline. Other potential risk factors, including age, body height, body weight, BMI, smoking, knee pain, osteoporosis, radiographic calcification of abdominal aorta, work history, treatment history of diabetes mellitus, kidney disorder, liver disorder, gout, hypertension, thyroid disorder, tuberculosis and osteoporosis were not significantly associated with disc height narrowing.

**Table 4:** Disc height measurement

Variables	Odds ratio	95 % CIs	p-value
Female gender	2.59	1.14–4.76	0.0006
Knee OA	2.21	1.13–4.34	0.0197
Low back pain	2.11	1.20–3.69	0.0253

Disc height is expressed as the disc height index (DHI), which was calculated as: [(Ha + Hp)/Ds + Di] × 100

*Ha* anterior disc height, *Hp* posterior disc height, *Ds* superior disc depth, *Di* inferior disc depth

## Discussion

The results of this population-based cohort study showed that the lumbar disc height of the village inhabitants gradually decreased over ten years and did not differ significantly among disc levels. The results of this study indicate that female gender, radiographic knee OA and the presence of low back pain are significantly associated with risk for development of lumbar disc height narrowing in the elderly.

A definition of intervertebral disc degeneration has not been uniformly applied, although it is well known that disc tissue shows progressive morphological, structural, histological, biochemical and functional changes with aging [25, 26]. In the early stage of degeneration, the water content of the hydrostatic nucleus pulposus, as shown by magnetic resonance imaging (MRI), decreases and microstructural changes within the disc tissues, such as clefts and tears, are often identified [27–29]. With progression, more severe morphological changes, such as anulus tears, disc prolapse and end-plate damage, are seen, with the formation of osteophytes [27]. In this advanced stage, disc height narrowing becomes evident on lumbar radiographs; this change is referred to as radiographic lumbar spondylosis [30], which has the potential to progress to such degenerative spinal diseases as lumbar spine canal stenosis [10, 11]. Therefore, although disc height narrowing is the most commonly used specific finding to indicate disc degeneration in the clinical setting, this change should be considered to represent an advanced stage of disc degeneration with significant structural changes.

In the study reported here, radiographic lumbar disc height was manually measured using a previously reported method of Farafan’s measurements [22, 23] with modifications. Amonoo-Kuofi [31] radiographically measured human IVD height on cross-sections and found that disc height increased until the fifth decade of life, after which there was an appreciable decline. To date, we are aware of no studies that report the quantitative assessment of disc height in a population-based longitudinal study of the elderly. The results of our study show a 5.8 % decrease in the disc height index of the lumbar spine over ten years with no significant differences in the rate of decrease among the disc levels.

The occurrence pattern of multilevel disc height narrowing has previously been evaluated based on the classification of multilevel disc degeneration [24, 32], which contains contiguous and skipped patterns. In our study, the percentage of subjects with multilevel severely degenerated (SD) discs was 45 % of all subjects. Among these, the percentage of subjects with skipped SD was 53 %; this percentage was much higher than that of subjects with degenerated discs in the Southern Chinese cohort study (21 %) as reported by Cheung and colleagues [24]. Different from this previous cross-sectional MRI-based study reported by Cheung and colleagues [24], our study evaluated the change in disc height by radiographic measurements in a longitudinal study (an average of 8.7 years follow-up). These differences could account for the large difference in the percentage of the subjects with skipped multilevel disc degeneration.

There have been a few longitudinal studies of risk factors associated with intervertebral disc degeneration. Elfering et al. [14] conducted a longitudinal MRI investigation of lumbar disc degeneration, and found that disc herniation, the lack of athletic activities, and night-shift work are significant risk factors for the development of lumbar disc degeneration. In a population-based longitudinal study of the progression of lumbar disc degeneration, Hassett et al. [15] reported that progression of disc space narrowing, semi-quantitatively assessed by lumbar radiography, was predicted by age, back pain, and radiographic hip and knee OA. Valdes et al. [17] reported a candidate gene association longitudinal study, and found that genes regulating inflammatory pathways [including matrix metalloproteinase-3 (MMP-3), tissue inhibitor of metalloproteinase 1 (TIMP-1), and cyclooxygenase-2 (COX-2)] were associated with radiographic progression of lumbar disc degeneration.

The results of our study showed that female gender is a risk factor for disc height narrowing in the elderly. This is in accordance with the findings of de Schepper et al. [33], who reported that the frequency of radiographic disc space narrowing was significantly higher in women than men in a cross-sectional cohort population aged 55 years and older. Muraki et al. [30] examined the incidence and risk factors for radiographic lumbar spondylosis assessed by Kellgren and Lawrence (K-L) grades [30], and found the incidence of disc space narrowing is higher in women. An MRI-based cohort study in the elderly by Wang et al. [34] also found female subjects had more severe disc degeneration. More recently, Wang et al. [35] reported that the prevalence and severity of disc space narrowing is higher in elderly women than in elderly men in a large population study. Recent evidence increasingly suggests that sex hormones influence the severity of disc degeneration [36]. Estrogens are known to have diverse physiological actions (e.g., on growth and proliferation) in several organs, including the intervertebral disc [37, 38], and estrogen deficiency is a factor contributing to accelerated disc degeneration [36, 39]. These studies, and ours, suggest the possibility that being a postmenopausal woman is one of the risk factors for progression of disc degeneration.

In agreement with the study results of Hasset [15], the results of our study showed that radiographic OA is a risk factor associated with disc height narrowing. Cross-sectional studies have suggested a relationship between disc degeneration and generalized OA [40, 41]. Biochemically, it is well known that the components and metabolism of the extracellular matrix of intervertebral discs and articular cartilage share common characteristics. Recent advances in genetic studies suggest that certain genetic polymorphisms may be relevant to development of degenerative musculoskeletal disorders, such as OA and disc degeneration (see review [42]). It has recently been suggested that genetic polymorphisms associated with knee OA [e.g., in asporin [43, 44] and growth differentiation factor 5 [45, 46]] may also be related to disc degeneration [47].

Hassett et al. [15] found that having back pain at baseline had a significant association with the progression of disc height narrowing. We also found a significant association of back pain with progressive disc height narrowing. Although the pathogenesis of back pain in the elderly is not fully understood, lumbar spondylotic changes, including disc height narrowing [30] and spinal alignment changes with lumbar spondylosis [48], are thought to have an effect on low back pain. The results of our risk factor analysis suggest that the existence of low back pain furthered the progression of lumbar spondylotic changes, including disc height narrowing in the elderly.

In our study of an older population, BMI had no association with disc height narrowing. However, the effect of being overweight on disc degeneration is controversial. In two extensive population-based longitudinal studies [14, 15], no significant relationship between high BMI and disc degeneration was identified. On the other hand, in a cross-sectional MRI-based study of adolescents and young adults, Samarzis et al. [49] reported that juvenile disc degeneration was strongly associated with being overweight. Liuke et al. [50] also reported an association between being overweight and lumbar disc degeneration in a population-based MRI study in middle-aged men, and provided evidence that a BMI above 25 kg/m^2^ increased the risk of disc degeneration, with a greater effect at younger ages than in middle age. This suggests that high BMI is strongly associated with disc degeneration, especially in the young. Therefore, it may be appropriate to interpret our finding that BMI has no association with development of disc height narrowing in the elderly with caution.

Although the mechanism of disc degeneration remains largely unknown, it is generally believed that progression of disc degeneration is attributable to degradation of IVD tissues by environmental factors, such as mechanical stimuli and injury, and/or intrinsic factors, such as compromise of local nutrition to the disc, the aberrant expression of cytokines and cell death, under the influence of heredity and genetic factors [25, 42, 51–53]. Our study focused on the progression of radiographic disc height narrowing in commonly seen degenerative lumbar diseases in an elderly population at least 65-years-old. The results of our study indicate that, under the influence of heredity and genetic factors [42, 51, 53], gender and/or local factors associated with aging may play a role in disc height narrowing, which is representative of the progression of degenerative structural changes in IVD tissues.

This study has several limitations. First, the subjects of this study were limited to a minimum age of 65 years-old. Therefore, the risk factors identified in this study cannot be regarded as those for the general population. Second, the follow-up period was not the same for all subjects, ranging from 6–10 years, thus possibly introducing heterogeneity into the analysis of DHI data.

## Conclusions

The lumbar disc height of the elderly inhabitants of a mountain village gradually decreased over time, and the rate of change in disc height did not differ significantly among disc levels. A risk factor analysis using a multivariate logistic regression model showed that female gender, radiographic knee OA and having low back pain are significantly associated with development of lumbar disc height narrowing in the elderly. The progression of disc degeneration is one of the underlying pathologies of the degenerative lumbar diseases commonly seen in an elderly population. More comprehensive epidemiologic research evidence on disc degeneration would contribute not only to understanding its pathology, but would also provide clues for a prophylactic approach and future treatment of the diseases associated with disc degeneration.
